# Proteomic alteration in gastic adenocarcinomas from Japanese patients

**DOI:** 10.1186/1476-4598-5-75

**Published:** 2006-12-25

**Authors:** Takahiro Yoshihara, Yoshito Kadota, Yoshiyuki Yoshimura, Yutaka Tatano, Naohiro Takeuchi, Hiroshi Okitsu, Atsushi Umemoto, Takashi Yamauchi, Kohji Itoh

**Affiliations:** 1 Department of Medicinal Biotechnology, Institute for Medicinal Resources, Graduate School of Pharmaceutical Sciences, The University of Tokushima, 1-78 Sho-machi, Tokushima 770-8505, Japan; 2 Department of Biochemistry, Graduate School of Pharmaceutical Sciences, The University of Tokushima, 1-78 Sho-machi, Tokushima 770-8505, Japan; 3 Department of Surgery, Graduate School of Medicine, The University of Tokushima, 1-78 Sho-machi, Tokushima 770-8505, Japan

## Abstract

**Background:**

Gastric adenocarcinomas comprise one of the common types of cancers in Asian countries including Japan. Comprehensive protein profiling of paired surgical specimens of primary gastric adenocarcinomas and nontumor mucosae derived from Japanese patients was carried out by means of two-dimensional gel electrophoresis (2D-EP) and liquid chromatography-electrospray ionic tandem mass spectrometry (LC-ESI-MS) to establish gastric cancer-specific proteins as putative clinical biomarkers and molecular targets for chemotherapy.

**Results:**

Relatively common alterations in protein expression were revealed in the tumor tissues. Increases in manganese dismutase and nonhistone chromosomal protein HMG-1 (HMG-1) were observed, while decreases in carbonic anhydrases I and II, glutatione-S-transferase and foveolin precursor (gastrokine-1) (FOV), an 18-kDa stomach-specific protein with putative tumor suppressor activity, were detected. RT-PCR analysis also revealed significant down-regulation of FOV mRNA expression in tumor tissues.

**Conclusion:**

A possible pathological role for down-regulation of FOV in gastric carcinogenesis was demonstrated. Evaluation of the specific decreases in gene and protein expression of FOV in patients may be utilized as clinical biomarkers for effective diagnosis and assessment of gastric cancer.

## Background

Gastric adenocarcinomas comprise one of the common types of cancers in Asian countries including Japan, being second only to lung cancer as to the number of deaths it causes. In spite of the recent development of diagnostic techniques, most gastric cancer patients are diagnosed at an advanced stage and have a very low five-year survival rate (less than 10%) [1]. This is partially due to a lack of specific and sensitive biomarkers for the diagnosis and monitoring of disease progress at an early stage, although some gastric tumor markers, including the carcinoembryonic antigen, have been used and are partly effective. As gastric carcinogenesis is a multistep process, comprehensive analysis is also required for individual cases, in which different molecular events occur in each carcinogenic process.

Recently, proteomic analysis was utilized to comprehensively examine protein expression in bodily fluids, tissues and cells [2-4]. This approach, as clinical proteomics, is very useful for identifying disease-associated proteins that show changes in expression and modification corresponding to a disease condition [5,6]. These disease-related proteins are expected to be biomarkers for diagnosis and putative targeted proteins for treatment [7-9]. On the other hand, comprehensive analyses of transcriptomes in tumor tissues from various cancer patients using DNA microarrays and gene chips have been performed in recent years [10]. However, a lack of correlation between changes in mRNAs and carcinogenesis has been demonstrated, and quantitative and qualitative changes of post-translationally modified proteins as final gene products are considered to be more informative than those of mRNAs in tumor tissues for studying the molecular events in carcinogenesis. Proteomic studies for the identification of tumor-associated proteins in gastric cancer are increasing, and proteome databases for gastric tissues [11] and cell lines [12] have been constructed. Most of them concern specific proteins or antigens that reflect the chemo- and thermo-resistant properties of stomach cancer [13-15], and that are associated with *Helicobactor pylori *[16,17]. In the present study, we performed comprehensive proteome analysis of tumor and nontumor tissues in Japanese patients with gastric carcinomas, and identified several proteins of which the expression levels are commonly altered in clinical cases. In particular, the expression of gastrokine-1 (GKN-1) was suggested to be under both transcriptional and translational control.

## Results

### Protein separation and identification

**Figure 1:**
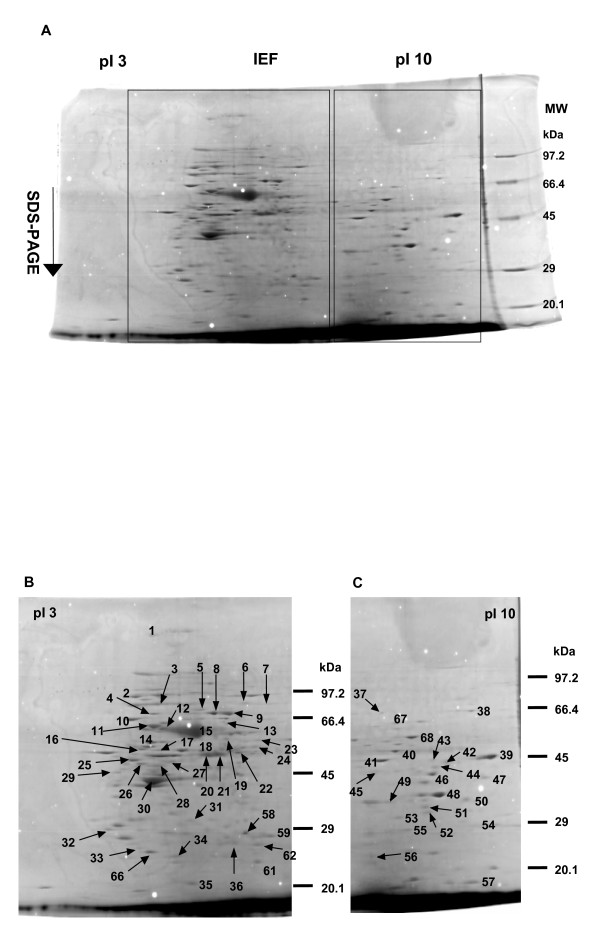
(A) An overview of a master 2D gel image for tumor tissue derived from a patient with a gastric adenocarcinoma. (B) and (C) The numbered protein spots were identified by LC-ESI-MS/MS and protein matching, as shown as enlarged figures.

Figure 1A shows an image overview of a typical master gel for a gastric tumor tissue. Around 200 protein spots stained with Coomassie brilliant blue (CBB) R-250 were well separated in the gels. The numbered spots in Figure 1B and 1C were excised from a gel, treated with trypsin and then subjected to liquid chromatography-electronic spray ionization tandem mass spectrometer (LC-ESI-MS/MS) analysis. Seventy-two of them representing 69 different protein species were identified. Table 1 lists all of the proteins identified through peptide matching with the Mascot search algorithm. The accuracy in protein profiling was evaluated as the score value (above 37).

**Table 1:** Protein profile detected in tumor tissue derived from a Japanese patient with a gastric adenocarcinoma.

Spot No.	Accession number	Protein identification	Mass	S.C. (%)	pI	Score
1	24308169	axonemal heavy chain dynein type 3	473776	0	6.04	37
2	15010550	heat shock protein gp96 precursor	90309	7	4.73	62
3	72222	heat shock protein 90-β	83584	14	4.97	176
4	5729877	heat shock 70 kDa protein 8 isoform 1	71082	19	5.37	283
5	38044288	gelsolin isoform b	80876	5	5.58	63
6	4826960	glutaminyl-tRNA synthetase	88655	1	6.71	44
7	4501867	aconitase 2	86113	10	7.36	231
8	119717	ezrin	69470	7	5.94	75
9	4557871	transferrin	79280	9	6.81	97
10	16507237	heat shock 70 kDa protein 5	72402	15	5.07	165
11	24234686	heat shock protein 70 kDa protein 8	53598	16	5.62	160
12	4885431	heat shock 70 kDa protein 1B	70267	11	5.48	143
13	1082886	tumor necrosis factor type 1 receptor-associated protein TRAP-1	75694	13	8.43	288
14	31542947	chaperonin, mitochondrial matrix protein P1, P60 lymphocyte protein	61187	13	5.7	299
15	28592	serum albumin	71316	23	6.05	292
16	340219	vimentin	53738	18	5.03	79
17	4503729	similar to FK506-binding protein 4	49031	5	5.6	36
18	32709	IFP53	53559	21	5.93	277
19	4502643	chaperonin-containing TCP1, subunit6A (zeta 1)	58444	6	6.23	79
20	7106439	tubulin, β5	50095	14	4.78	125
21	37492	α-tubulin	50810	13	5.02	94
22	125604	pyruvate kinase, M2 isozyme	58447	5	7.95	234
23	4557014	catalase	59947	6	6.9	120
24	4503483	eukaryotic translation elongation factor 2	96246	4	6.41	56
25	179279	ATP synthase β subunit	56861	5	5.39	59
26	7657041	down-regulated in metastasis	320846	2	7.07	40
27	4504169	glutathione synthetase, GSH synthetase	52523	5	5.67	99
28	12017952	GE36	73327	2	5.2	37
29	34234	laminin-binding protein	31888	12	4.84	51
30	16359158	actin, beta	42078	14	5.29	171
31	6635125	KIAA0284 protein	161473	2	6.36	47
32	5803225	14-3-3 epsilon	29326	8	4.63	49
33	4504707	inositol polyphosphate-4-phosphatase, type II, 105 kD	105749	1	5.87	37
34	35038601	hypothetical protein DKFZp761A078	74864	2	7.27	47
35	12017952	GE36	73327	1	5.2	38
36	4505877	plectin 1 isoform 1	520111	1	5.57	44
37	38158018	centrosomal protein 1, centriole associated protein, centriolin	269874	1	5.44	39
38	30157438	CTD-binding SR-like protein rA9	180240	1	9.15	41
39	4503471	eukaryotic translation elongation factor 1 α1	50451	12	9.1	191
40	4757810	ATP synthase	59828	17	9.16	178
41	693933	2-phosphopyruvate-hydratase α-enolase	47421	22	7.01	240
42	123576	47 kDa heat shock protein precursor	46352	14	8.27	177
43	5032069	splicing factor 3b, subunit 4	44414	3	8.54	58
44	4503471	eukaryotic translation elongation factor 1 α1	50451	4	9.1	47
45	5921789	citrate synthase, mitochondrial precursor	51959	12	8.13	114
46	4505763	phosphoglycerate kinase 1	44985	14	8.3	87
47	4504069	aspartate aminotransferase 2 precursor	47844	6	9.14	52
48	7669492	glyceraldehyde-3-phosphate dehydrogenase	36201	13	8.57	49
49	4757756	annexin A2	38808	9	7.57	87
50	6648067	malate dehydrogenase, mitochondrial precursor	35965	7	8.92	60
51	5031857	lactate dehydrogenase A	36950	6	8.44	43
52	238427	porin 31 HM	30737	29	8.63	124
53	5174447	guanine nucleotide-binding protein	35511	8	7.6	65
54	34740329	heterogeneous nuclear ribonucleoprotein A3	39799	7	9.1	43
55	4504983	galectin-3	26229	12	8.58	42
56	4504447	heterogeneous nuclear ribonucleoprotein A2/B1 isoform A2	36041	7	8.67	40
57	86901	ATP-dependent DNA helicase RAP30/74 chain RAP30	26350	3	9.46	41
58	230445	carbonic anhydrase I	28903	12	6.44	67
59	455739	carbonic anhydrase II	29285	8	6.87	82
60	26892090	beta-globin chain variant	16101	40	7.86	121
61	34709	manganese superoxide dismutase	24891	10	8.35	111
62	4507645	triosephosphate isomerase 1	26938	17	6.45	47
63	38488935	foveolin precursor	20546	16	5.65	95
64	478813	nonhistone chromosomal protein HMG-1	25139	13	5.41	60
65	2204207	glutathione S-transferase	23595	34	5.43	73
66	178755	proapolipoprotein	28944	8	5.45	103
67	37267	transketolase	68435	4	7.9	59
68	189998	M2-type pyruvate kinase	58447	10	7.95	157
69	825605	glutathione S-transferase	25650	10	8.51	65

These proteins can be classified into several categories based on their functions, including cytoskeleton proteins, stress-related and chaperoning proteins, acute-phase proteins, glycolytic enzymes, enzymes involved in metabolism and cell proliferation, tumor suppressor proteins and stomach-specific proteins.

### Common alterations of protein expression between tumor and nontumor tissues in gastric cancer patients

**Figure 2:**
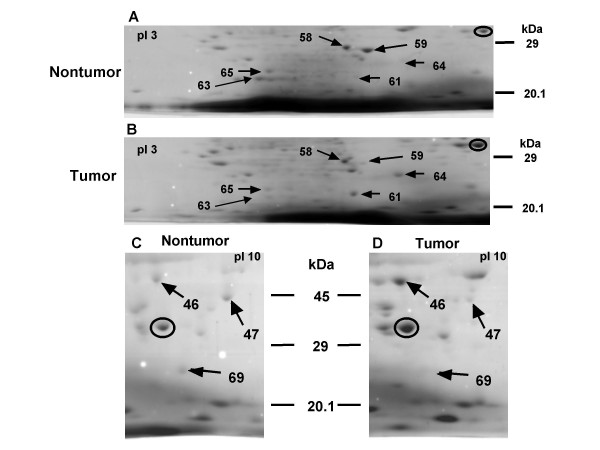
Detailed alteration patterns of proteins. (A) Nontumor and (B) tumor proteins including CAI (No.58), CAII (No.59), MnSOD (No.61), FOV (No.63), HMG-1 (No.64), and GST (No.65). (C) Nontumor and (D) tumor proteins including PGK-1 (No.46), AST (No.47), and GST (No.69). GAPDH, circled, was used as a reference protein.

Diverse alterations in proteomes were detected between tumor and nontumor tissues from the same patients. As shown in Figure 2, the several common alterations were observed among in five Japanese gastric cancer patients (Cases A to E). Manganese superoxide dismutase (MnSOD), nonhistone chromosomal protein HMG-1 (HMG-1), phosphoglycerate kinase 1 (PGK-1), carbonic anhydrase I and II (CA I and II), foveolin precursor FOV (gastrokine-1), aspartate aminotransferase 2 precursor (AST), and glutathione S-transferase (GST) exhibited common changes in expression between tumor and nontumor tissues, including among the identified proteins. The protein expression of MnSOD and HMG-1 was demonstrated to be up-regulated in tumor tissues compared to in nontumor tissues. On the other hand, the CA I and II, FOV, AST and GST proteins were revealed to be down-regulated in tumor tissues. The fold changes in the expression of these proteins relative to that of GAPDH are summarized in Table 2. The most remarkable decrease was shown in the level of FOV in all cases. The degree of the decrease in GST protein expression was almost the same as that in FOV (Cases B, D, and E), although one was not observed in the other two cases. On the other hand, the increase in HMG-1 was marked in three cases (A, C, and D), although such a difference in expression was not observed in the other two cases. MnSOD exhibited a tendency to increase in the tumor tissues of three patients (Cases A, C, and D), but a relative decrease was also observed in Case B. As for PGK-1, a significant relationship between fold changes in protein expression and the pathological grading of tumors was hardly observed.

**Table 2:** Fold changes in protein expression between nontumor and tumor tissues derived from five patients with gastric adenocarcinomas.

Protein	Case A	Case B	Case C	Case D	Case E
CA I	-6.5	-1.6	-3.3	-4.3	-2.6
CA II	-2.6	-1.3	-26	-4.3	-13
FOV	-13	-13	-26	*	-26
MnSOD	+1.7	-2.6	+6.5	+1.4	+1.1
HMG-1	+13	*	+3.7	+13	*
PGK1	+1.6	+1.1	-1.2	+2.6	-1.4
AST	-13	-1.6	-6.5	-3.7	-2.6
GST	*	-26	*	-13	-13

CA I, carbonic anhydrase I.

CA II, carbonic anhydrase II.

FOV, foveolin precursor.

MnSOD, manganese superoxide dismutase.

HMG-1, nonhistone chromosomal protein.

PGK1, phosphoglycerate kinase 1.

AST, aspartate aminotransferase 2 precursor.

GST, glutathione S-transferase.

*, not determined.

### RT-PCR analysis

**Figure 3:**
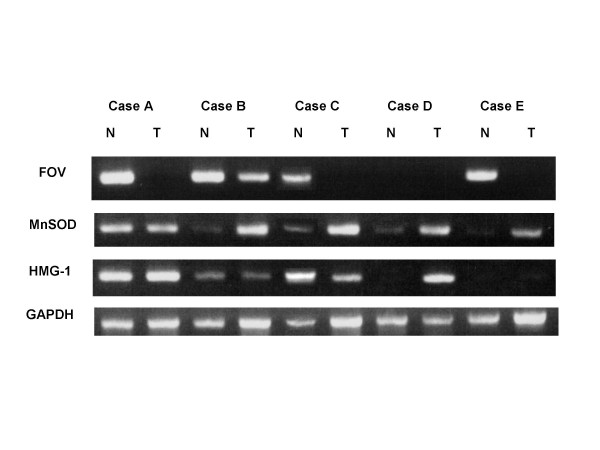
Comparison of mRNA expression of proteins possibly associated with carcinogenesis between nontumor and tumor tissues derived from five patients with gastric adenocarcinomas (cases A to E) by RT-PCR. N and T indicate nontumor and tumor tissues, respectively.

By RT-PCR, changes in mRNA expression in tumor and nontumor tissues derived from gastric cancer patients were analyzed for proteins that exhibited alterations in protein expression, including FOV, MnSOD and HMG-1. As shown in Figure 3, FOV mRNA was significantly decreased in tumor tissues in four at all patients (Cases A, B, C, and E), while it was not detected in either nontumor or tumor tissues from the other patient (Case D). On the other hand, MnSOD mRNA was markedly increased in four patients (Cases B, C, D, and E), although little difference in the mRNA level between nontumor and tumor tissues in Case A was detected. However, a relationship between HMG-1 mRNA expression and the pathological phenotypes of tumors was hardly observed.

## Discussion

In this study we performed proteomic analysis of tumor and nontumor tissues derived from Japanese gastric adenocarcinoma patients. Sixty-nine different proteins in the tumor tissue from a gastric cancer case were identified by 2D-EP and LC-ESI-MS/MS, which included stress proteins, Hsp70, Hsp90 and chaperonine-containing TCP1 (CCT), for self-protection; glycolytic enzymes, triose phosphate isomerase 1, α-enolase and PGK-1, for the growing energy requirement; cytoskeletal proteins, ezrin, gelsolin isoform b and vimentin; proteins involved in cell differentiation and proliferation, galectin-3 and transferrin; and proteins exhibiting putative tumor suppressor activity, FOV. Many of these proteins have been reported to be associated with the tumorigenesis of gastric adenocarcinomas involving multiple steps and factors [18,19].

We also demonstrated that the expression levels of several proteins in tumor tissues were commonly altered in five Japanese patients with gastric adenocarcinomas, compared to those in nontumor tissues. A common increase in protein expression in tumor tissues occurred for MnSOD, HMG-1 and PGK-1, whereas a common decrease in tumor tissues occurred for FOV, GST and AST.

Superoxide (O_2_^-^), a free radical, is essential for the anti-microbial action of granulocytes and monocytes. Superoxide dismutase (SOD) rapidly removes the excess amount of superoxide produced in the stress and biological reactions *in vivo *through catalysis of the conversion of superoxide with H^+ ^to H_2_O_2 _and O_2_. MnSOD, one of the SODs, is located in mitochondria and contributes to the protection of mitochondrial DNA from damage. It has been reported that enhanced expression of MnSOD in progressive gastric cancer should be related to the 5-year survival rate after surgery [20] and sensitivity to chemotherapy [21]. In this study, protein expression of MnSOD was up-regulated in 4 of 5 gastric cancer patients, suggesting a self-protecting response. On the other hand, the up-regulation of MnSOD in tumor tissues has been considered to interfere with effective chemotherapy based on radical production, which may cause a decrease in sensitivity to anti-cancer drugs and determine the severity of the cancer.

HMG-1 regulates the transcription of various genes and the structural stabilization of chromosomes as a DNA-binding protein. HMG-1 has been reported to be associated with carcinogenesis and metastasis in colorectal and breast cancer [22].

Transcriptional up-regulation of HMG-1 in gastric cancer has also been demonstrated [23]. Expression of HMG-1 in tumor tissues was suggested to be related to resistance to cisplatin [24]. We demonstrated here an increase in this protein in three cases.

GST is a drug-metabolizing enzyme that catalyzes the conjugation of reduced glutathione to drugs and metabolites, and also contributes to the detoxification of carcinogens. Therefore, a decrease in the activity may be a risk factor for carcinogenesis. Infection by Helicobacter pylori has been reported to cause a decrease in GST expression [25], suggesting that a decrease in GST activity might be a cause of gastric carcinogenesis. In this study, a decrease in GST protein expression was observed in three cases. A decrease in GST protein expression may be utilized as a biomarker for the diagnosis of gastric tumors.

AST is an aminotransferase that acts on amino acids and α-keto acid, that is distributed widely in human organs. AST is released into the blood stream due to enhanced permeability and destruction of tissues. A elevated concentration of AST protein has been reported in the blood of patients with hepatitis, malignant tumors including hepatomas, and leuchemia. Gene expression of GST has also been demonstrated to be up-regulated in colorectal tumor tissues [26]. However, a decrease in AST protein expression was commonly observed in all tumor tissues derived from the present five patients with gastric adenocarcinomas. Further study should be performed to elucidate whether a decrease in the AST protein is specifically observed in gastric tumor tissues or not.

Elevated expression of PGK-1, which is one of the enzymes in the glycolytic pathway and which catalyzes the dephosphorylation of 1,3-bisphosphoglycerate to produce ATP, has been observed in many malignant tumor tissues that are dependent on ATP as a major energy source. Solid tumors are thought to need the overproduction of ATP to maintain the enhanced proliferation. The up-regulation of glycolytic enzymes, including PGK-1 in lung cancer [27] and M2-type pyruvate kinase in colorectal cancer [28], has been reported and suggested to be useful for cancer screening. However, in the present study, a significant relation between PGK-1 protein expression and the phenotypes of gastric adenocarcinomas was hardly detected. Therefore, PGK-1 was considered to be not suitable for the diagnosis of gastric cancer.

Carbonic anhydrases (CAs) are zinc-containing enzymes that are distributed widely in various organs and that comprise a large family including CA-I to CA-IX. They catalyze the hydration of CO_2 _for intermediate metabolism, and maintain the pH and ion equilibrium in the body. So far a direct relationship has been demonstrated between malignant transformation and protein expression for CAs I through VII [29]. Two earlier studies revealed that the expression of both CA-I and CA-II was significantly reduced in colorectal tumors compared to in normal colorectal epithelia or mucosae [30]. Another report presented results showing that reduced expression of CA-I and CA-II was correlated with the biological aggressiveness of colorectal cancer and synchronous distant metastasis [31]. As suggested previously [32], gastric and colorectal carcinomas may share a similar mechanism of cell proliferation and mucosal malignancy, and may become a biomarker for these carcinomas, because common decreases in the protein expression of CA-I and CA-II were also observed in this study.

FOV, which is identical to a stomach-specific 18-kDa antrum mucosa protein! (AMP-18) [33,34], GKN1 [35], and trefoil factor interactions(z) (TFIZ) [36], was demonstrated to be dramatically down-regulated or absent in the gastric adenocarcinoma patients in this study. The human AMP-18 and human carcinoantigenic CA11 gene product, which encodes an amino acid sequence that differs from that of AMP-18 in only a single residue [37,38], function as secreted growth factors partly responsible for maintaining a normal functional gastric epithelium [33]. Both AMP-18 and the CA11 gene product have been reported to be intensively expressed in normal stomach tissue but not in most gastric cancers [33,37,38]. Immunohistochemical studies demonstrated that AMP-18 appears to be present in mucosal epithelial cells of the normal human gastric antrum and duodenum [33]. Recently, TFIZ was demonstrated to act as a growth regulator of gastric epithelial cells through the formation of a heterodimer with trefoil factor 1 (TFF1) [36]. Our present data further confirmed the high expression of FOV in nontumor gastric tissues and the significant suppression of the protein in gastric adnocarcinomas, indicating that FOV play roles in the maintenance of normal differentiation of epithelial cells and tumor suppression but not in tumor tissues. Furthermore, we demonstrated that transcription of FOV mRNA was also commonly down-regulated in gastric cancer patients, indicating that a marked decrease in FOV protein was caused by suppression of FOV gene expression. Furthermore, in one patient the protein was not detected in nontumor tissues, suggesting that the expression level of FOV protein in individuals may determine the gastric adenocarcinoma phenotype. Accordingly, the expression level of FOV as a biomarker may be informative for the assessment of gastric cancer.

## Conclusion

Protein profiling of tumor and nontumor tissues derived from Japanese patients with gastric adenocarcinomas was performed using 2D-EP and LC-ESI-MS/MS. The identified proteins included molecular chaperones, energy-producing enzymes, cytoskeletal proteins, and so on. Common protein alterations were detected in the gastric cancer patients. Protein expression of MnSOD and HMG-1 was up-regulated while that of GST, AST and FOV was down-regulated in gastric tumor tissues. A correlation between the alteration of these proteins and their transcriptional expression in gastric cancer was hardly observed in this study, except for in the case of FOV. Both the protein and gene expression of FOV, a stomach-specific secretory growth factor for normal gastric epithelial cells, was markedly down-regulated in tumor tissues derived from Japanese patients with gastric adenocarcinomas. Monitoring of the expression levels of this stomach-specific protein in clinical samples may provide useful information for the diagnosis of gastric cancer as a specific biomarker and for better understanding of gastric carcinogenesis.

## Methods

### Materials

DNase I, RNase A, 2-mercaptoethanol (2-ME), glass beads (212–300 μm), Nonidet P-40, acrylamide, *N,N,N',N'*-tetramethylenediamine (TEMED), sodium dodecylsulfate (SDS), iodoacetamide and dithiothreitol (DTT) were purchased from Sigma (St. Louis, MO). Agarose for isoelectronic focusing (IEF), and Pharmalyte pI 3–10, 4–6.5 and 8–10.5 were from Amersham Bioscience (Piscataway, NJ). Trypsin (sequencing grade) was from Roche (Manheim, Germany). Phenylmethylsulfonyl fluoride (PMSF), thiourea, sorbitol, sodium pyrophosphate, ammonium persulfate, D,L-aspartic acid, trichloroacetic acid, sulfosalicylic acid dihydrate, acetic acid, acetonitrile, formic acid and trifluoroacetic acid (TFA) were from Wako Pure Chemicals (Osaka, Japan). Urea was from Katayama Chemicals (Osaka, Japan). Pepstatin A and leupeptin were from the Peptide Institute (Osaka, Japan). NH_4_HCO_3 _and N,N'-methylenebisacrylamide were from Nacalai Tesque (Kyoto, Japan). CBB R-250 was from ICN Biomedicals Inc. (Aurora, OH). Molecular mass standards were from APRO Science, Inc. (Tokushima, Japan). TRIZOL reagent was from Life Technologies (Frederick, MD). Oligo(dT)12–18 primer, deoxynucleotides (dNTPs), and RNaseOUT were from Invitrogen (Carlsbad, CA). M-MLV reverse transcriptase and Taq DNA polymerase were from Promega (Madison, WI).

### Tissues and sample preparation

Primary gastric adenocarcinomas and adjacent nontumor mucosae were collected on gastrectomy and provided by the Dept. of Surgery, Graduate School of Medicine, The University of Tokushima, Tokushima, Japan. The research was carried out in accordance with the Declaration of Helsinki of the World Medical Association, and was approved by the ethical committee of the University of Tokushima. Informed consent was also given by all of the patients who provided the clinical samples. Tissues were frozen in a dry ice-methanol bath as soon as possible after dissection and stored in a deep freezer (-80°C) before use. For mRNA analysis, tissues were first immersed in RNAlater (Takara, Tokyo, Japan) before freezing. Detailed clinicopathological data including the tumor stage (according to the AJCC system), site and differentiation, and histological data on the tissue samples are listed in Table 3. None of these cases were classified in the scirrhous type category, and tumor tissue was clearly distinguishted from non-tumor one in each case. For two-dimensional gel electrophoresis (2D-EP), protein extraction from tissues was carried out by the following procedure. Frozen blocks (20–30 mg wet weight) were homogenized with a plastic pestle (Toyobo, Tokyo, Japan) in the presence of glass beads in 10 vol/wet weight of dialysis buffer comprising 5 M urea, 1 M thiourea, 10 mM NaPPi, 1.67 μL/mL 2-ME, 0.005% DNase I, 0.05 mg/ml RNase A, 20 μM leupeptin, 1 mM EDTA, 2 mM PMSF and 20 μM pepstatinA, and then centrifuged at 50,000 rpm for 30 min at 4°C (Beckman -Coulter, Fullerton, CA). The resultant supernatant was used as the tissue extract. Protein concentrations were determined with a Bradford protein assay kit (Bio-Rad, Hercules, CA) using bovine gamma-globulin as a standard.

**Table 3:** Clinical features of the patients with gastric adenocarcinomas.

Case	Age	Sex	Location^a^	Grade^b^	Stage	
A	63	Male	UM	G3	IIIA	T2N2M0H0
B	68	Female	L	G2	II	T2N2M0
C	76	Male	ML	G2	IV	T4N3M0
D	78	Female	L	G2	III	T2N0M0H0
E	77	Male	ML	G2	II	T2N1M0

^a^U: upper, M: middle, L: lower

^b^G2: moderately differentiated, G3: poorly differentiated.

### Two-dimesional gel electrophoresis

2D-EP was carried out as described previously [39]. The first-dimensional isoelectric focusing was performed in an 1% (w/v) agarose gel (ϕ 2.6 × 180 mm) with a pH 3–10 gradient at 700 V for 18 hr at 4°C, and the second-dimensional SDS gel electrophoresis was performed with a 5–15% (w/v) acrylamide gradient (*M*r range, 6–200 kDa) in a standard slab gel (20 × 13 cm) at 15 mA for 3 h, and then at 70 mA for 2 h at room temperature. Gels were stained with CBB R-250.

Protein samples (500 μg) extracted from the tumor center and surrounding histologically normal mucosa were subjected to 2D-EP and run in pairs side by side.

Some of the stained spots were excised from the 2D-gel, in-gel digested with trypsin and then subjected to LC-ESI-MS/MS analysis as described previously [40]. The peptide mixture was separated with a reversed phase nanoLC system (Famous, Swichos II, Ultimate, LC Packings, Sunnyvale, CA). The eluted peptides were sprayed directly into an ESI mass spectrometer (Esquire3000 Plus, Bruker-Daltonics, Fremont, CA).

A large volume of MS/MS data was acquired with DataAnalysis 3.1 software (Bruker-Daltonics), converted to text files listing the mass values of the parent ions, and intensities and masses of fragment ions, and then processed with the MASCOT algorithm (Matrix Science Ltd, London, U.K.) to assign peptides in the NCBI non-redundant sequence database using a taxonomic restriction, 'human'. The database search was performed with the parameters described by Yoshimura et al. [40].

### Image analysis and MS peptide sequencing

Image acquisition and analysis were performed with Molecular Imager FXProPlus (Bio-Rad) and ImageMaster software (Bio-Rad). Comparisons were made between gel images of tumor and matched nontumor samples pair by pair. Normalized volume differences were statistically calculated for all five cases. The content of glyceraldehyde 3-phosphodehydrogenase (GAPDH) protein was used as a reference to evaluate the fold alteration of protein expression between tumor and matched nontumor tissues as the levels of GAPDH mRNA and protein were not changed between tissues derived from patients. Consistently and significantly different spots were selected for analysis by LC-ESI-MS/MS. Protein spots!were cut out from gels in small pieces, and subjected to in-gel tryptic digestion overnight [40]. Peptide mass spectra were recorded, and parameters for spectra acquisition were used as stated previously [40]. Accuracy in database protein matching using Mascot Search [41] was judged as a score over 37, which was obtained in most of the analyses.

### RNA isolation and RT-PCR analysis

Tumor and matched nontumor samples (approx. 50 mg wet weight) were minced, and then homogenized manually in 1 ml of TRIZOL reagent (Invitrogen) on ice. RNA was isolated without DNase I treatment according to the manufacturer's protocol. Briefly, 0.2 ml of CHCl_3 _was added to the homogenate, followed by centrifugation at 20,600 × g for 15 min. An equal volume of 2-propanol was added to the resultant supernatant to precipitate RNA. After centrifugation, the pellet was rinsed with 75% ethanol/diethylpyridylchloride (DEPC)-treated water, followed by drying. The pellet was dissolved in an appropriate volume of DEPC-treated water as the total RNA fraction. For reverse transcription (RT), 2 μg of RNA from each sample was transcribed at 37°C for 1 h in the presence of 200U of Molony leukemia virus reverse transcriptase (Promega), oligo(dT)_12–18 _primer, 0.5 mM dNTPs and 50U of RNaseOUT. The PCRs for carbonic anhydrase-I and II, glutatione-S-transferase, FOV, and GAPDH were performed within a linear range of amplification using the selected primer set and conditions, and expected size of products, as summarized in Table 4. The PCR products were analyzed by 1.5% agarose gel electrophoresis and stained with ethidium bromide.

**Table 4:** Primer sets used for RT-PCR analysis.

Primers		Primer sequences
MnSOD	sense	5' - acgcggcctacgtgaacaacctgaa - 3'
	antisense	5' - aaccccaacctgagccttggacacc - 3'
HMG-1	sense	5' - cgggaggagcataagaagaagcacc - 3'
	antisense	5' - caatggacaggccaggatgttctcc - 3'
GAPDH	sense	5' - gtcatccatgacaactttgg - 3'
	antisense	5' - tgctgtagccaaattcgttg - 3'

MnSOD, manganese superoxide dismutase.

HMG-1, nonhistone chromosomal protein.

GAPDH, glyceraldehyde-3-phosphate dehydrogenase.

## Abbreviations

2D-EP, Two-dimensional gel electrophoresis; 2-ME, 2-Mercaptoethanol; AMP-18, Antrum mucosa protein-18; AST, Aspartate aminotransferase 2 precursor; CA I and II, Carbonic anhydrase I and II; CAs, Carbonic anhydrases; CBB, Coomassie brilliant blue; CCT, Chaperonine-containing TCP1; DEPC, Diethylpyridylchloride; dNTPs, Deoxynucleotides; DTT, Dithiothreitol; FOV, Foveolin precursor (gastrokine-1); GAPDH, Glyceraldehyde 3-phosphodehydrogenase; GKN1, Gastrokine-1; GST, Glutathione S-transferase; HMG-1, Nonhistone chromosomal protein HMG-1; IEF, Isoelectronic focusing; LC-ESI-MS/MS, Liquid chromatography-electronic spray ionization-tandem mass spectrometer; MnSOD, Manganese superoxide dismutase; PGK-1, Phosphoglycerate kinase 1; PMSF, Phenylmethylsulfonyl fluoride; RT, Reverse transcription; SDS, Sodium dodecylsulfate, SOD, Superoxide dismutase; TEMED, *N,N,N',N'*-Tetramethylenediamine; TFA, Trifluoroacetic acid; TFF1, Trefoil factor 1; TFIZ, Trefoil factor interactions(z).

## Competing interests

The author(s) declare that they have no competing interests.

## Authors' contributions

TY mainly carried out the molecular studies, participated in the proteomic determination and RT-PCR analysis. YK carried out the proteomic studies, partly participated in the proteomic determination. YY supported the proteomic studies by TY. YT partly participated in the RT-PCR for gastric cancer-related protein. NT parly participated in the proteomic determination. HO carried out the operation of gastric cancer patiens, participated in providing the cancer tissues. AU carried out the operation of gastric cancer patients, participated in providing clinical information. TY supported the proteomic studies and advised the methods. KI conceived of the study, and participated in its design and coordination. All authors read and approved the final manuscript.
